# Platelets of Healthy Origins Promote Functional Improvement of Atherosclerotic Endothelial Progenitor Cells

**DOI:** 10.3389/fphar.2019.00424

**Published:** 2019-04-24

**Authors:** Nicoleta Alexandru, Florentina Safciuc, Alina Constantin, Miruna Nemecz, Gabriela Tanko, Alexandru Filippi, Emanuel Dragan, Elisabeta Bãdilã, Adriana Georgescu

**Affiliations:** ^1^Institute of Cellular Biology and Pathology ‘Nicolae Simionescu’ of the Romanian Academy, Bucharest, Romania; ^2^Internal Medicine Clinic, Emergency Clinical Hospital, Bucharest, Romania; ^3^‘Carol Davila’ University of Medicine and Pharmacy, Bucharest, Romania

**Keywords:** platelets, late endothelial progenitor cells, atherosclerosis, animal model, mir-223

## Abstract

The purpose was to evaluate the effect of platelets on functional properties of late endothelial progenitor cells (EPCs), in the direct co-culture conditions, and to investigate the involved mediators, in experimental induced atherosclerosis. The late EPCs obtained from two animal groups, hypertensive-hyperlipidemic (HH) and control (C) hamsters, named late EPCs-HH and late EPCs-C, were co-incubated with or without platelets isolated from both groups. Our results have showed that exposure to platelets from control animals: (i) promoted the late EPCs-C capacity to form colonies and capillary-like structures, and also to proliferate and migrate; (ii) improved the functional properties of late EPCs-HH; (iii) strengthened the direct binding EPCs-platelets; (iv) increased SDF-1α,VEGF, PDGF, and reduced CD40L, IL-1β,-6,-8 levels; and (v) enhanced miR-223 and IGF-1R expressions. Platelets from HH group diminished functional abilities for both EPC types and had opposite effects on these pro-angiogenic and pro-inflammatory molecules. Furthermore, testing the direct effect of miR-223 and IGF-1R on late EPCs disclosed that these molecular factors improve late EPC functional properties in atherosclerosis in terms of stimulation of the proliferation and migration abilities. In conclusion, *in vitro* exposure to platelets of healthy origins had a positive effect on functional properties of atherosclerotic late EPCs. The most likely candidates mediating EPC-platelet interaction can be SDF-1α, VEGF, CD40L, PDGF, IL-1β,-6,-8, miR-223, and IGF-1R. The current study brings evidences that the presence of healthy origin platelets is of utmost importance on functional improvement of EPCs in atherosclerosis.

## Introduction

Endothelial progenitor cells (EPCs) represent a heterogeneous cell population derived from circulating CD34 positive or CD34 and KDR/VEGF receptor-2 (KDR/VEGFR2) double positive MNCs (Zhang et al., 2013). Previous studies demonstrated that two different types of circulating EPCs, the early and the late EPCs, can be derived and identified from peripheral blood (Hirschi et al., 2008). Early EPCs are an angiogenic EPC population obtained from short-term cultures of 4–7 days, *in vitro*. These cells form colony forming units (CFUs) and possess many endothelial characteristics, such as harboring CD31, TIE2, and VEGFR2 markers (Asahara et al., 1997). Late EPCs, called also out-growth EPCs, have different growth patterns and are usually obtained from long-term cultures of at least 2–3 weeks, *in vitro*. Late EPCs possess in addition to early EPC specific markers, other endothelial characteristics, such as VE-cadherin and vWF (Shantsila et al., 2007). These cells can further differentiate into mature endothelial cells and are capable of forming new blood vessels through a process of vasculogenesis (Lee and Poh, 2014).

Endothelial dysfunction has been associated with the development of atherosclerosis and cardiovascular diseases, and maintaining balance between endothelial injury and recovery is critical for reducing cardiovascular events (Shantsila et al., 2007). Since mature endothelial cells possess a limited regenerative capacity, in recent years has increased the focus on circulating EPC study, as they may maintain endothelial integrity, function and postnatal neovascularization (Lee and Poh, 2014). Thus, significant *in vitro* and *in vivo* studies have established that EPCs play an important role in vascular repair and regeneration (Shi et al., 1998; Takahashi et al., 1999; Urbich and Dimmeler, 2004). In addition, it has been reported that EPCs can be a prognostic marker for cardiovascular events, since a significant correlation between various cardiovascular risk factors, cardiovascular disease states and reduced EPC levels and function has existed (Lee and Poh, 2014; Raz et al., 2014). Furthermore, it has been reported that late EPCs can home to areas of injury and integrate into damaged vessels, suggesting their role in the vascular repair (Zhao et al., 2012).

There are evidences that the EPC involvement in vascular injury repair is mediated by their interaction with platelets. It has been showed that platelets have a significant involvement into EPC recruitment to sites of vascular injury, and in their maturation and differentiation to endothelial cells (Daub et al., 2006; de Boer et al., 2006; Langer et al., 2006; Lev et al., 2006; Massberg et al., 2006; Leshem-Lev et al., 2010). Moreover, *in vitro* studies have demonstrated that an essential interplay occurs between activated platelets and EPCs under both static and flow conditions (de Boer et al., 2006; Langer et al., 2006; Lev et al., 2006; Raz et al., 2014). Apart from these evidences, the effects of platelets on EPC functionality have been investigated only in a few studies. Thus, it has been showed that platelets enhance human early EPC capacity to proliferate, migrate, form colonies, to express endothelial markers and produce NO metabolites (Leshem-Lev et al., 2010). Recently, it has been established that the positive effects of platelets on EPCs can be mediated, at least in part, by factors they secreted, such as PDGF (Raz et al., 2014). In our previous study *in vivo*, we demonstrated in a hypertensive-hypercholesterolemic hamster (HH) model, that circulating EPC administration suppresses the development of atherosclerosis and reduces hepatic lipid and macrophage accumulation with the consequent alleviation of dyslipidemia and hypertension (Alexandru et al., 2015). Moreover, we postulated that EPCs modulate proinflammatory pathways in the vasculature, and platelet microparticles (PMPs) might influence EPC homing and vascular repair. If platelets have a direct contribution to EPC homing to sites of damaged endothelium, this has remained a question that we could not answer at that time. As a result, the study was designed to evaluate *in vitro* the interplay between platelets and late EPCs and to investigate the mediators responsible for the effect of platelets on EPC functional properties, in experimental induced atherosclerosis.

## Materials and Methods

### The Generation of Diet-Induced Hypertensive-Hyperlipidemic Murine Model to Study Atherosclerosis, the Source for Late EPC Cultures

Male Golden Syrian hamsters (3 months old, *n* = 24) were divided into two equal groups: (1) HH (simultaneously hypertensive-hyperlipidemic hamsters) fed with an ordinary chow enriched with 3% cholesterol, 15% butter for hyperlipemia and 8% NaCl for hypertension, for 4 months; (2) C (control hamsters), age-matched normal healthy animals which were kept in the same housing conditions and received classical food containing 1% NaCl, for 4 months (Alexandru et al., 2011, 2013, 2015, 2017a; Georgescu et al., 2012, 2013, 2016; Andrei et al., 2014). After 4 months of diet, the peripheral blood was collected from the retro-orbital plexus from the animals in both experimental groups to obtain late EPC cultures. In addition, we performed a second experiment with new hamsters of the same age and sex and divided the same experimental groups to obtain platelets. This experiment was offset by a month, the time required to obtain late EPC cultures (as per the procedure).

### Culture of Murine Late EPCs

The experiments were accomplished according to methods designated by Medina et al. (2012) and Alexandru et al. (2017a). Late EPCs-C have been got from MNCs from peripheral blood of C group, and late EPCs-HH from MNCs from peripheral blood of HH group. More details regarding the isolation and characterization of late EPCs were presented at Supplementary Material.

### Platelet Isolation

For this step, hamsters were slightly anesthetized with 2% isoflurane in oxygen (2.4 l/min), and blood was collected from the retro-orbital plexus. Platelets were separated, from both groups, according to the method reported by Leshem-Lev et al. (2010) and Alexandru et al. (2011, 2013). Platelets isolated from C group were called PLTs-C, and those from HH group, PLTs-HH. The platelets were counted at Gallios flow cytometer, and adjusted at the same number (2 × 10^6^ platelets) for all co-culture experiments.

### Co-culture of Platelets With Late EPCs

Late EPCs (3 × 10^4^) obtained in culture after 28 days, as described above, were co-incubated with or without platelets (2 × 10^6^/500 μl medium with 1% antibiotics penicillin, neomycin and streptomycin), in 5% CO_2_ atmosphere, at 37°C, for 7 days, into 4-well plates. The late EPCs-C co-incubated with PLTs-C were named late EPCs-C+PLTs-C, and those incubated with PLTs-HH, late EPCs-C+PLTs-HH. In the same manner, we called the late EPCs-HH co-incubated with PLTs-C or PLTs-HH, late EPCs-HH+PLTs-C and late EPCs-HH+PLTs-HH, respectively. For all culture or co-culture experiments, EGM-2 medium without supplements and 10% FBS was used. After 7 days in culture, co-culture conditioned medium was transferred to a microtube, centrifugated at 2500 *g* for 15 min and kept at −80°C for ELISA measurements. After removing the medium, the cell functions were analyzed. In distinct experiments, after medium removal, the 500 μl Trizol^®^ LS reagent was added for 10 min, and late EPCs-C, late EPCs-HH, late EPCs-C+PLTs-C, late EPCs-C+PLTs-HH, late EPCs-HH+PLTs-C, late EPCs-HH+PLTs-C in Trizol were kept at −80°C until RNA extractions.

### Platelet and EPC Labeling

The PLTs-C and PLTs-HH (2 × 10^6^), isolated as described above, were labeled with 2 × 10^−6^ M PKH26 dye (a red fluorescent aliphatic chromophore) for 5 min at room temperature (RT). Then, the platelets were centrifuged at 2000 rpm for 15 min, at 20°C, suspended in EBM-2 without supplements and FBS, and incubated with 3 × 10^4^ late EPCs-C or late EPCs-HH, for 7 days, as follows: late EPCs-C with PLTs-C or PLTs-HH, and late EPCs-HH with PLTs-C or PLTs-HH. After the staining of EPC nuclei with DAPI solution, the samples were observed under an inverted fluorescent microscope (Axio Vert.A1 Fl, Carl Zeiss, software Axio Vision Rel 483SE64-SP1).

### Colony Forming Unit Assays for Late EPCs

In our experiments, CFUs for the late EPCs were counted at 7 days after plating on collagen-coated wells whatever the presence or absence of platelets according to the protocol described by Leshem-Lev et al. (2010), using an inverted microscope. Results were given as the mean number of CFUs per well.

### EPC Proliferation Assay

The MTT assay was performed to investigate the proliferation of late EPCs-C or late EPCs-HH alone or co-incubated with PLTs-C or PLTs-HH conformed with the protocol described by Alexandru et al. (2017a).

### EPC Migration Assay

*In vitro* migration of late EPCs-C or late EPCs-HH (at 4 weeks) incubated with or without platelets for 7 days was evaluated by using a Boyden chamber assay as previously described (Yu et al., 2011; Alexandru et al., 2017a). The migrated cells were counted by independent blinded researchers, and the mean cell number was quantified at 10× magnification.

### *In vitro* Angiogenesis Assay of EPCs

The capillary formation was analyzed after plated the cells on Matrigel, according to the manufacturer’s procedure. Therefore, 30 μl Matrigel, melted overnight at 4°C on ice, were added into well of a pre-cooled 96-well plate and afterward, incubated at 37°C for 60 min. The late EPCs-C and late EPCs-HH (at 4 weeks) incubated with PLTs-C or PLTs-HH for 7 days were washed three time with PBS to remove platelets, and after trypsinization with 0.25% trypsin were counted. Then, the 6 × 10^4^ late EPCs-C or late EPCs-HH were resuspended into 100 μl EBM-2-EGM-2+10% FBS medium and plated onto this 96-well plate pre-incubated with Matrigel, for 24h. The tube formation was viewed under an inverted microscope.

### Analysis of SDF-1α, VEGF, PDGF, CD40L, and IL-1β,-6,-8 Levels

The measurements were performed on cell culture medium, collected from late EPCs-C and late EPCs-HH, after incubation or not with platelets using commercially available ELISA kits according to the manufacturer’s instructions (R&D Systems; cat # DSA00; Minneapolis, MN, United States). Briefly, samples were added in each well of a 96-well microtiter plate coated with antibodies, all specific for investigated chemokines, and incubated for 1 or 2 h at RT. After washing, adding of conjugate, substrate and stop solution, the optical density at 450 nm was measured using a spectrophotometer (TECAN, InfiniteM200PRO, Austria).

### Assessment of miR-223 Expression

The miR-223 expressions in cultures of late EPCs-C, late EPCs-HH, before and after the incubation with PLTs-C or PLTs-HH as follows: late EPCs-C+PLTs-C, late EPCs-C+PLTs-HH, late EPCs-HH+PLTs-C and late EPCs-HH+PLTs-HH, were quantified by RT q-PCR (real-time quantitative-PCR) as described previously by Schmittgen and Livak (2008) and Alexandru et al. (2017a).

### Immunohistochemical Detection of Insulin-Like Growth Factor 1 Receptor Expression

The thin deparaffinized sections (7 μm) incubated with IGF-1R antibody [IGF-1R Antibody (N-20): sc-712, from SantaCruz], at 4°C overnight, were washed with 1% BSA into PBS for 15 min, three times, incubated with secondary antibody conjugated to FITC [anti-rabbit IgG (whole molecule) - FITC antibody, F0382, from Sigma], at RT for 1 h, and mounted with Fluoroshield with DAPI before fluorescence microscopy examination.

### Transfection of miR-223 in Late EPCs

To study the direct biological effects of miR-223 on late EPC function, the miR-223 transfection was performed in late EPCs using Lipofectamine 2000 (Invitrogen) accordance to the manufacturer’s protocol. Therefore, late EPCs-C and late EPCs-HH were planted at a density of 6 × 10^4^ cells/well in EBM-2-EGM-2+10% FBS medium and transfected with 100 nmol/l miR-223 (Zhu et al., 2013) for 72 h. The RT-qPCR was used to evaluate the transfection efficiency by quantitating the miR-223 expression in cells before and after transfection. Subsequently, the experiments of proliferation and migration were performed on both late-EPCs-C and late-EPCs-HH.

### Incubation of Late EPCs With Recombinant IGF-1R Protein

To investigate *in vitro* direct IGF-1R effects, the late EPCs-C and late EPCs-HH were planted at a density of 6 × 10^4^ cells/well in EBM-2-EGM-2+10%FBS medium and co-incubated with recombinant IGF-1R protein (5 pmol) for 1h, and afterward the proliferative and migratory abilities of the cells were analyzed. The Western Blot was used to quantify the protein expression of IGF-1R as a measure of its efficient incubation with late EPCs.

## Results

### Animal Models and Late EPC Cultures

The control (C) and atherosclerotic (HH) hamsters characterized in our previous papers (Alexandru et al., 2011, 2013, 2015, 2017a; Georgescu et al., 2012, 2013, 2016) were the source for obtaining late EPC cultures, late EPCs-C and late EPCs-HH, respectively, characterized by Dil-acLDL/UEA-1 uptake and the presence of endothelial lineage markers (C133, CD34, KDR, CD144, Tie-2, vWF, CD45, and CD14) as described by Alexandru et al. (2017a).

To investigate whether interplay between platelets and late EPCs is essential for improving EPC functional properties, we directly co-incubated the cultured late EPCs with platelets, both from C or HH groups, on the same plate in separately experiments. After co-incubation with platelets, late EPCs were supplementary characterized by following the expression of specific surface receptors. Immunofluorescence detection of CD34, KDR, C133, CD144, vWF, Tie-2, CD14, and CD45 by flow cytometry revealed similar percentages to those found at late EPCs alone, meaning that the cells after incubation still maintain their ability to differentiate into mature cells as described by Alexandru et al. (2017a).

### Evidence for Direct Interaction of Platelets With Late-EPCs

**FIGURE 1:**
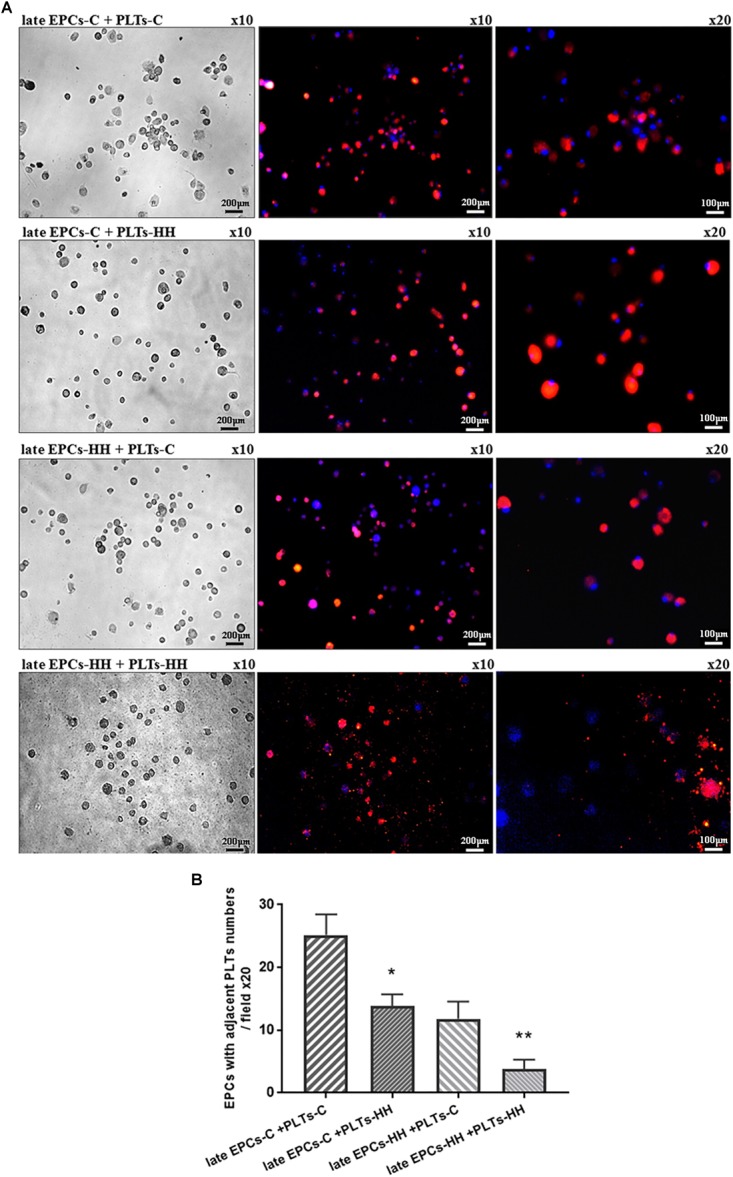
**(A)** Representative images of late EPCs from C (late EPCs-C) and HH (late EPCs-HH) animals, stained with DAPI and incubated with platelets isolated from C group (PLTs-C) and HH group (PLTs-HH), respectively, marked with PKH26. Left panel: phase contrast microscopy images; Right panel: fluorescence images with late EPCs in blue and platelets in red. **(B)** The quantification of adherent PLTs-C or PLTs-HH to late EPCs-C and late EPC-HH. The five independent experiments have been considered for mean and standard deviation (SD) (SD). The statistically significant differences between the groups were calculated, and represented as ^∗^*P* ≤ 0.05 for values late EPCs-C+PLTs-HH vs. late EPCs-C+ PLTs-C and as ^∗∗^*P* ≤ 0.05 for values late EPCs-HH+PLTs-HH vs. late EPCs-HH+ PLTs-C.

In an attempt to demonstrate the direct interaction of platelets with late EPCs, we incubated late EPCs-C or late EPCs-HH stained with DAPI solution with platelets from C (PLTs-C) or HH (PLTs-HH) groups labeled with PKH26, for 7 days (Figure 1). It could be seen that the binding of PLTs-C to either late EPCs-C or late EPCs-HH was higher compared to that observed at PLTs-HH (Figure 1A). EPCs and platelets adjacent to each other were quantified by florescence microscopy. Compared to adherent PLTs-C, the number of adherent PLT-HH to late EPCs-C is by 1.83 times smaller (^∗^*P* ≤ 0.005, Figure 1B) and by 3.10 times smaller to late EPCs-HH (^∗∗^*P* ≤ 0.005, Figure 1B). The results suggest that physical contact of platelets with late EPCs is affected in atherosclerotic conditions.

**FIGURE 2:**
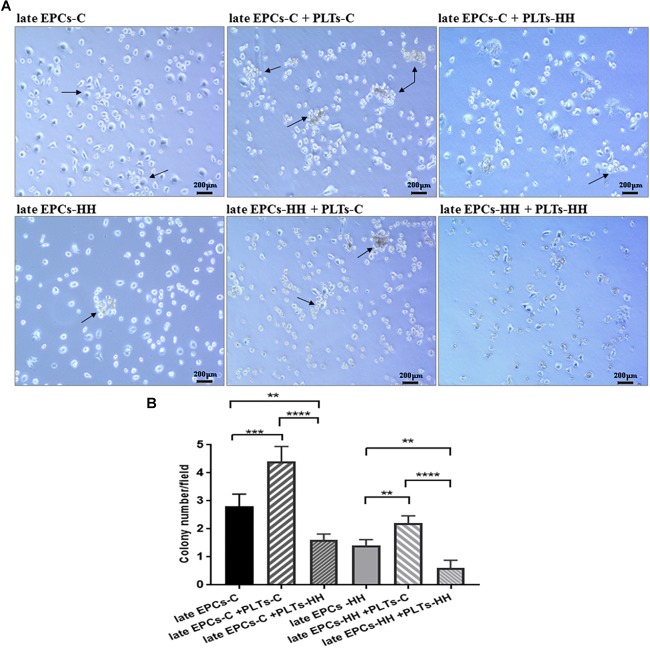
**(A)** Representative EPC colonies after 7 days of co-incubation with isolated platelets from C (PLTs-C) and HH (PLTs-HH) groups, compared with EPCs alone (on collagen type I) (magnification was ×10). **(B)** The quantification of the number of EPC colonies (CFUs) in the absence and presence of platelets. The five independent experiments have been considered for mean and SD, and CFUs were counted in at least three randomly chosen visual fields. The statistically significant differences between the groups were calculated using One-way ANOVA followed by Bonferroni’s multiple comparison test, one pair being compared with late EPCs-C and the other one with late EPCs-HH, and represented as ^∗∗^*P* ≤ 0.01, ^∗∗∗^*P* ≤ 0.005, and ^∗∗∗∗^*P* ≤ 0.001.

### Colony Forming Units of EPCs in the Presence of Platelets

The first step to follow the effects of direct interaction of platelets with late-EPCs on EPC functional properties was to investigate the formation of CFUs (Figure 2).

Consequently, co-incubation of the either late EPCs-C or late EPCs-HH (grown on collagen type I) with PLTs-C for 7 days generated a higher number of CFUs compared with late EPCs alone: 4.2 ± 0.28 CFUs/well for late EPCs-C incubated with PLTs-C vs. 2.8 ± 0.37 CFUs/well for late EPCs-C alone (*n* = 6, ^∗∗∗^*P* ≤ 0.005, Figure 2A,B), and 2.3 ± 0.17 CFUs/well for late EPCs-HH incubated with PLTs-C vs. 1.3 ± 0.145 CFUs/well for late EPCs-HH alone (*n* = 6, ^∗∗^*P* ≤ 0.01, Figure 2A,B).

In addition, co-incubation of either late EPCs-C or late EPCs-HH with PLTs-HH had a negative effect inducing a reduction of CFU number after 7 days of culture: 1.6 ± 0.24 CFUs/well for late EPCs-C incubated with PLTs-HH vs. 2.8 ± 0.37 CFUs/well for late EPCs-C alone (*n* = 6, ^∗∗^*P* ≤ 0.01, Figure 2A,B), and 0.6 ± 0.218 CFUs/well for late EPCs-HH incubated with PLTs-HH vs. 1.3 ± 0.145 CFUs/well for late EPCs-HH alone (*n* = 6, ^∗∗^*P* ≤ 0.01, Figure 2A,B). Furthermore, co-incubation of either late EPCs-C or late EPCs-HH with PLTs-C for 7 days induced an enhances of CFU number comparing to their co-incubation with PLTs-HH: 4.2 ± 0.28 CFUs/well for late EPCs-C incubated with PLTs-C vs. 1.6 ± 0.24 CFUs/well for late EPCs-C incubated with PLTs-HH (*n* = 6,^∗∗∗∗^*P* ≤ 0.001, Figure 2A,B), and 2.3 ± 0.17 CFUs/well for late EPCs-HH incubated with PLTs-C vs. 0.6 ± 0.218 CFUs/well for late EPCs-HH incubated with PLTs-HH (*n* = 6,^∗∗∗∗^*P* ≤ 0.001, Figure 2A,B).

### Platelets Stimulate the Functional Properties of EPCs

In order to further investigate the effects of platelets on functional aspects of the cultured EPCs, the proliferative and migratory capacities have been examined during atherosclerosis in a *in vitro* model.

#### Platelets Influence the Proliferation of EPCs

**FIGURE 3:**
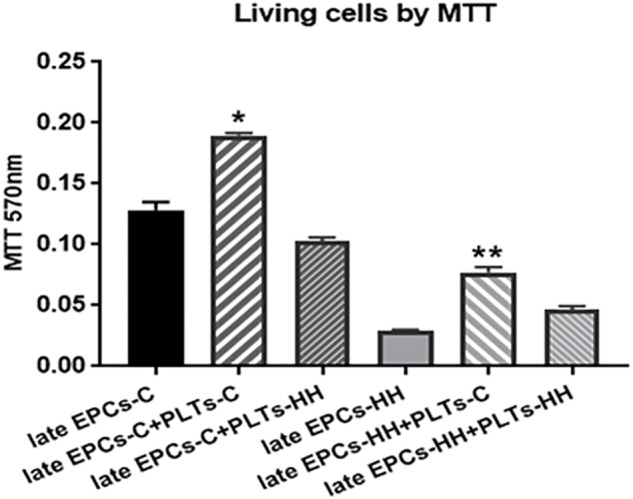
MTT proliferation assay following co-incubation of late EPCs with platelets from C group (PLTs-C) or platelets from HH group (PLTs-HH), vs. late EPCs alone. Co-incubation with PLTs-C induced a rise of number of living cells. The five independent experiments have been considered for mean and SD. The statistically significant differences between the groups were calculated, and represented as ^∗^*P* ≤ 0.05 values vs. late EPCs-C, and as ^∗∗^*P* ≤ 0.05 values vs. EPCs-HH.

Co-incubation of either late EPCs-C or late EPCs-HH with isolated PLTs-C induced a rise of number of living cells when it was compared with late EPCs alone: the increase was by ∼1.48-fold for late EPCs-C and by ∼ 2.71-fold for late EPCs-HH (^∗^*P* ≤ 0.005, Figure 3). The experiments were performed by the MTT viability assay. In parallel experiments, PLTs-HH were added to either late EPCs-C or late EPCs-HH in cultures. In contrast with PLTs-C, PLTs-HH were unable to significantly affect EPC proliferation. The OD values for late EPCs-C incubated with PLTs-HH were lower compared to the values took for late EPCs alone, indicating that the proliferation of late EPCs-C was slower in the presence of activated platelets (PLT-HH) (Figure 3). As for the OD values for late EPC-HH incubated with PLTs-HH, these were slightly increased compared to the values from late EPC-HH alone, but there is no statistically significant difference between groups (Figure 3).

#### Platelets Control Migratory Capacity of EPCs

**FIGURE 4:**
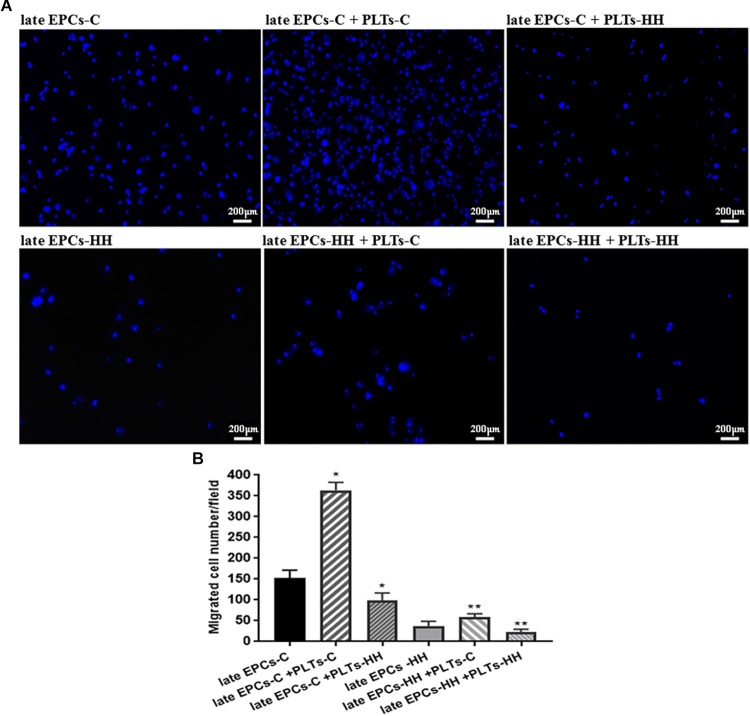
Migration analysis following EPC co-incubation with platelets from C group (PLTs-C) or with platelets from HH group (PLTs-HH), vs. EPCs alone. Migratory ability was assessed as number of migrated cells per 2 × 10^4^ cells added to the Boyden chamber. **(A)** Representative images with migrated late EPCs-C, late-EPCs-HH stained with DAPI and incubated with or without PLTs-c or PLTs-HH; **(B)** The quantification of transmigrated cells; the five independent experiments have been considered for mean, respectively SD, and cells were counted in at least three randomly chosen visual fields (magnification was × 10). The statistically significant differences between the groups were calculated, and represented as ^∗^*P* ≤ 0.05 values vs. late EPCs-C and ^∗∗^*P* ≤ 0.05 values vs. late EPCs-HH.

The effect of platelets on EPC function was further assessed *in vitro* by migration assays. The ability of late EPC to migrate was evaluated using a Boyden chamber, observing the transmigrated EPCs into the lower side of chamber. Transmigrated EPCs were quantified after DAPI staining by counting cells in six microscopic fields. The number of transmigrated late EPCs-HH was reduced (36.8 ± 3.28) compared to late EPCs-C number (152.25 ± 9.13) (*P* ≤ 0.01, Figure 4), suggesting a diminished migration capacity for late EPCs-HH. Addition of PLTs-C improved the migratory ability for both investigated cell types, the transmigrated cell number increasing by ∼2.39-fold for C cells, and 1.61-fold for HH cells (*P* ≤ 0.01, Figure 4). In marked contrast, these effects were not observable when adding PLTs-HH. In this situation the number of migrated late EPCs-C and EPCs-HH in the Boyden chamber decreased by 1.55-fold and 1.62-fold, respectively (*P* ≤ 0.05, Figure 4).

#### Platelets Promote EPCs to Form Tube-Like Structures

**FIGURE 5:**
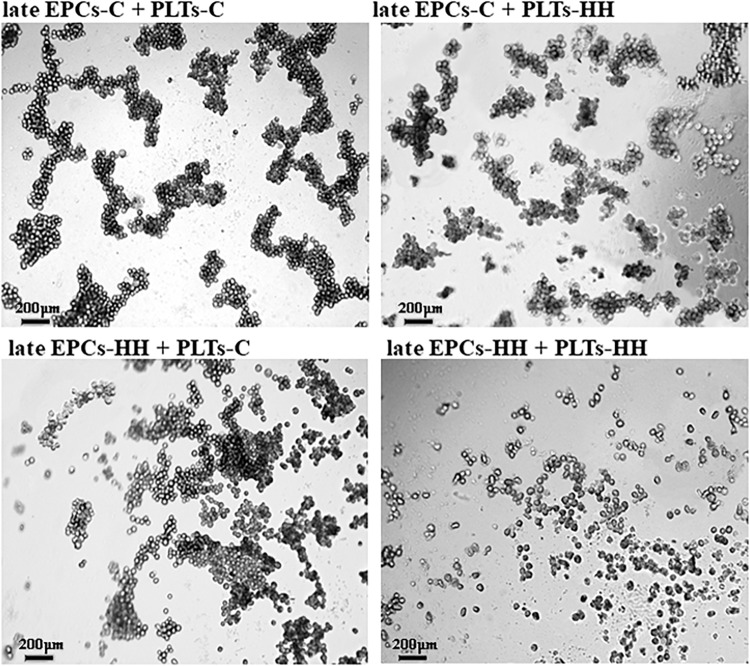
Representative images for capillary-like sprouts designed by late EPCs-C+PLTs-C, late EPCs-C+PLTs-HH, late EPCs-HH+PLTs-C and late EPCs-HH+PLTs-HH onto Matrigel.

The effect of platelets on EPC function was also assessed *in vitro* by angiogenesis assay. After incubation with PLTs-C, the late EPCs-C seeded on three-dimensional Matrigel extracellular matrix have been grouped into capillary-like sprouts, while late EPCs-HH had a lower ability to organize themselves into capillary-like structures (Figure 5). Incubation with PLTs-HH did not induce capillary-like tube formation capability of late EPCs-C or late EPCs-HH (Figure 5). The phase contrast microscopy was used to visualize the cells and the imagines were collected (×10 magnification), for four independent experiments per group.

### Highlighting the Mechanisms Underlying the EPC Functional Improvement Promoted by Healthy Origin Platelets

#### Chemokines and Factors Secreted Into Cell Culture Medium Mediate the Positive Effects of Platelets on Late EPC Functional Properties

In order to decipher the mechanisms involved in the interaction between platelets and EPCs the levels of several chemokines and factors, with role in inflammation and atherosclerosis process, were quantified into the culture medium collected after the 7 days of incubation.

Thus, the concentrations of SDF-1α, VEGF, CD40L, PDGF, and IL-1β,-6,-8 were measured in the medium obtained from late EPCs-C and late EPCs-HH incubated or not with PLTs-C or PLTs-HH for 7 days. We have found that SDF-1α, VEGF and PDGF-AB isoform levels were significantly higher in the presence of PLTs-C that being: by ∼1.23-fold, 1.11-fold, and 2.41-fold for late EPCs-C, and by ∼1.38-fold, 1.45-fold and 2.34-fold for late EPCs-HH, respectively (*P* ≤ 0.05, Table 1). In another set of experiments, the levels of CD40L, IL-1β, IL-6, and IL-8 were decreased after the incubation of late EPCs-C with PLTs-C, by ∼1.07-fold, 1.36-fold, 1.55-fold, and 1.13-fold, respectively, vs. late EPCs-C alone (*P* ≤ 0.05, Table 1). Concerning late EPCs-HH, the presence of PLTs-C generated a reduction of these molecule levels by 1.13-fold, 1.33-fold, 1.43-fold and 1.09-fold, respectively, comparative with late EPCs-HH alone (*P* ≤ 0.05, Table 1). Co-incubation with PLTs-HH induced a contrary effect on the levels of these pro-angiogenic and pro-inflammatory molecules secreted in the culture medium by both types of late-EPCs (Table 1).

**Table 1:** Chemokines and factors released into the culture medium after late EPC-platelet co-incubation for 7 days (*n* = 5 for each investigated molecule).

Chemokines/factors (pg/ml)	Late EPCs-C	Late EPCs -HH	Late EPCs-C+PLTs- C	Late EPCs-C+PLTs- HH	Late EPCs – HH+PLTs-C	Late EPCs-HH+PLTs- HH
SDF-1α	1345.17 ± 2.8	1240.12 ± 15.0	1660.56 ± 11.24	1081.2 ± 14.06	1492.5 ± 16.12	880.12 ± 10.1
		(^∗^*P* ≤ 0.05)	(^∗^*P* ≤ 0.05)	(^∗^*P* ≤ 0.05)	(^∗∗^*P* ≤ 0.05)	(^∗∗^*P* ≤ 0.05)
VEGF	139.22 ± 7.12	78.34 ± 5.37	154.32 ± 3.74	85.06 ± 5.30	123.40 ± 17.17	64.64 ± 4.37
		(^∗^*P* ≤ 0.05)	(^∗^*P* ≤ 0.05)	(^∗^*P* ≤ 0.05)	(^∗∗^*P* ≤ 0.05)	(^∗∗^*P* ≤ 0.05)
PDGF-AB	3.34 ± 0.99	1.84 ± 0.89	8.06 ± 1.05	2.69 ± 0.92	6.29 ± 0.90	1.25 ± 1.80
		(^∗^*P* ≤ 0.05)	(^∗^*P* ≤ 0.05)	(^∗^*P* ≤ 0.05)	(^∗∗^*P* ≤ 0.05)	
CD40L	464.94 ± 13.54	553.67 ± 2.39	433.89 ± 8.19	541.48 ± 10.66	491.27 ± 11.68	606.32 ± 14.21
		(^∗^*P* ≤ 0.05)	(^∗^*P* ≤ 0.05)	(^∗^*P* ≤ 0.05)	(^∗∗^*P* ≤ 0.05)	(^∗∗^*P* ≤ 0.05)
IL-1β	21.84 ± 6.26	34.088 ± 7.398	16.077 ± 2.863	29.49 ± 3.028	25.64 ± 2.32	41.48 ± 0.54
		(^∗^*P* ≤ 0.05)	(^∗^*P* ≤ 0.05)	(^∗^*P* ≤ 0.05)	(^∗∗^*P* ≤ 0.05)	(^∗∗^*P* ≤ 0.05)
IL-6	3.384 ± 0,34	5.361 ± 1.276	2.177 ± 0.221	4.312 ± 0.941	3.747 ± 0.461	7.482 ± 0.864
		(^∗^*P* ≤ 0.05)	(^∗^*P* ≤ 0.05)	(^∗^*P* ≤ 0.05)	(^∗∗^*P* ≤ 0.05)	(^∗∗^*P* ≤ 0.05)
IL-8	289.48 ± 14.58	335.67 ± 0.69	256.03 ± 7.94	316.93 ± 5.84	307.05 ± 8.96	353.8 ± 9.98
		(^∗^*P* ≤ 0.05)	(^∗^*P* ≤ 0.05)	(^∗^*P* ≤ 0.05)	(^∗∗^*P* ≤ 0.05)	(^∗∗^*P* ≤ 0.05)

*The comparisons were made with late EPC alone. The statistically significant differences between the groups were calculated, and represented as ^∗^P ≤ 0.05 values vs. late EPCs-C, and as ^∗∗^P ≤ 0.05 values vs. late EPCs-HH.*

#### Platelets Regulate EPC Functional Properties in a miR-223 and IGF-1R – Dependent Manner

**FIGURE 6:**
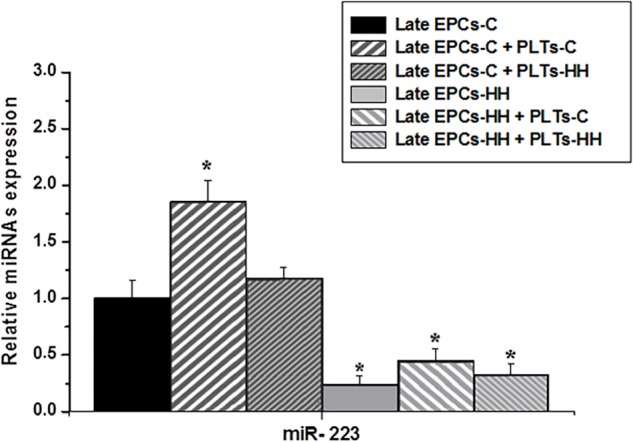
The analysis of miR-223 expression in cultures of late EPCs-C or late EPCs-HH co-incubated with or without platelets from C group (PLTs-C) or platelets from HH group (PLTs-HH) by RT-qPCR. The five independent experiments have been considered for mean and SD. The statistically significant differences between the groups were calculated, and represented as ^∗^*P* ≤ 0.05 values vs. late EPCs-C group.

The expression of miR-223, a miRNA that has an emerging role in inflammatory and metabolic disorders (Taibi et al., 2014), with a specific focus on atherosclerosis, was analyzed in late EPCs-C or late EPCs-HH before and after incubation with PLTs-C or PLTs-HH applying miRNA TaqMan assays. The results showed that miR-223 expression was significantly diminished in late EPCs-HH vs. late EPCs-C (*P* ≤ 0.05) (Figure 6). The presence of PLTs-C has led to a significant increase of miR-223 levels in late-EPCs-C and late-EPCs-HH, showing the platelet capability to transfer miR-223 into their target cells (*P* ≤ 0.001). On the other hand, PLTs-HH did not have any influence on miR-223 levels in late-EPCs-C or late-EPCs-HH (Figure 6).

Hence, these results indicate that the presence of healthy origin platelets is of utmost importance on functional improvement of EPCs in atherosclerosis by miR-223 transfer.

**FIGURE 7:**
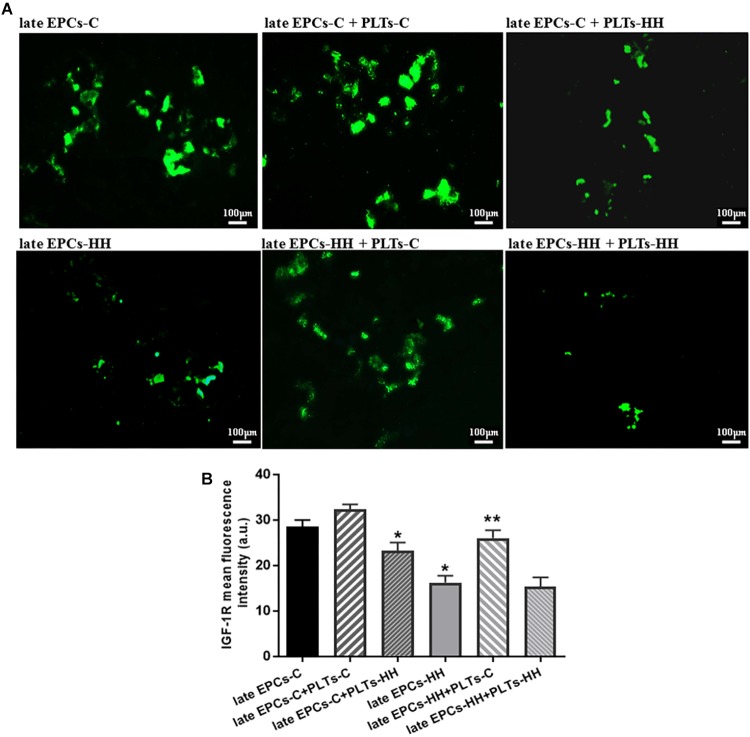
**(A)** The assessment of IGF-1R expression in late EPCs-C and late EPCs-HH incubated with or without PLTs-C or PLTs-HH, by the fluorescence microscopy (in green, magnification × 20). **(B)** The quantification of IGF-1R expression; the four independent experiments per group have been considered for mean, respectively SD. The statistically significant differences between the groups were calculated and represented as ^∗^*P* ≤ 0.05 for values vs. late EPCs-C and ^∗∗^*P* ≤ 0.05 for values vs. late EPCs-HH.

The role of insulin-like growth factor 1 (IGF-1R) on EPC function was further depicted by assessing its expression after platelets-EPC interaction. It has been already shown that, activation of the IGF-1R increases NO bioavailability systemically, resulting in improved EPC number and functions (such as proliferation and migration) (Fleissner and Thum, 2008). Also, knowing that IGF-1R is a functional target for miR-223 (Pan et al., 2014), we investigated the IGF-1R expression in cultures of late EPCs-C or late EPCs-HH incubated with or without PLTs-C or PLTs-HH, by immunofluorescence assay. The results revealed that the IGF-1R expression was decreased in late EPCs-HH compared to late EPCs-C, by ∼1.76-fold (*P* ≤ 0.05), and the addition of platelets of healthy origins enhanced it in both groups: by ∼1.13-fold for C cells, and 1.60-fold for HH cells (*P* ≤ 0.05) (Figure 7A,B). The incubation with PLTs-HH significantly reduced the IGF-1R expression in late EPCs-C, by ∼1.23-fold (*P* ≤ 0.05) (Figure 7A,B). When the PLTs-HH were added on late EPCs-HH for 7 day, the IGF-1R expression was slightly decreased, by ∼1.06-fold (Figure 7A,B).

These data help to decipher the mechanisms underlying the functional improvement of atherosclerotic late EPC supported by platelets of healthy origins: platelets deliver miR-223 into these cells (may be by microparticle release), and this one in turn targets IGF-1R improving late EPCs functions.

#### miR-223 and IGF-1R Directly Improve EPC Functional Properties

In order to investigate the direct effect of miR223 and IGF-1R on EPC function, *in vitro* proliferation and migration of late EPCs-C or late EPCs-HH transfected with miR-223 or incubated with recombinant IGF-1R protein in separate experiments were investigated.

**FIGURE 8:**
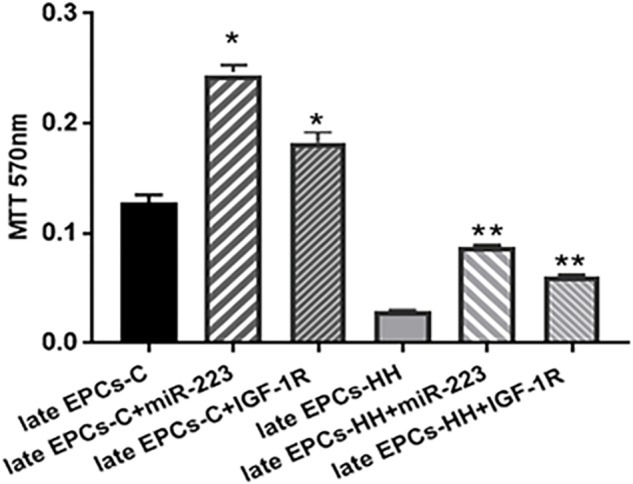
MTT proliferation assay following transfection of late EPCs with miR-223 (late EPCs-C + miR-223 and late EPCs-HH + miR-223) and co-incubation with IGF-1R (late EPCs-C + IGF-1R and late EPCs-HH + IGF-1R), vs. late EPCs alone. The five independent experiments have been considered for mean and SD. The statistically significant differences between the groups were calculated, and represented as ^∗^*P* ≤ 0.05 values vs. late EPCs-C and ^∗∗^*P* ≤ 0.05 values vs. late EPCs-HH.

The MTT viability assay has showed that transfection with miR-223 induced an increased proportion of living cells by ∼1.90-fold for late EPCs-C, and by ∼3.01-fold for late EPCs-HH (*P* ≤ 0.05, Figure 8), vs. late EPCs alone. The OD values for late EPCs-C and late EPCs-HH co-incubated with IGF-1R were also augmented compared to those from late EPCs alone, the enhance being by ∼1.42-fold for late EPCs-C, and by ∼2.08-fold for late EPCs-HH (*P* ≤ 0.05, Figure 8). These results suggested that both molecular factors, miR-223 and IGF-1R, increased the proliferation capacity of late EPCs.

**FIGURE 9:**
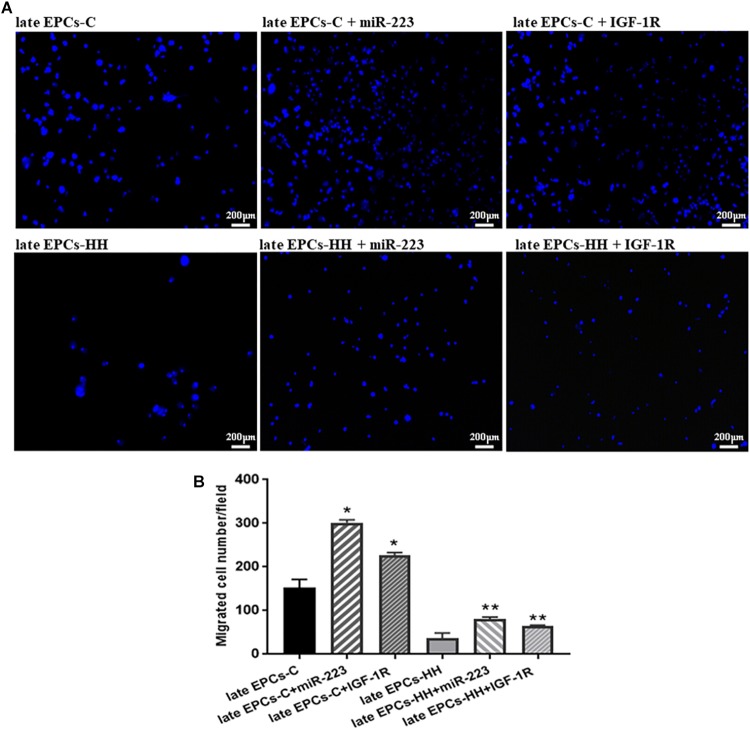
Migration assay following transfection with miR-223 or incubation with IGF-1R recombinant protein of late EPCs-C and late EPCs-HH. Migratory ability was assessed as number of migrated cells per 2 × 10^4^ cells added to the Boyden chamber. **(A)** Representative images with migrated late EPCs-C, late-EPCs-HH stained with DAPI and transfected with miR-223 or co-incubated with IGF-1R; **(B)** The quantification of transmigrated cells; The mean and SD of four independent experiments are shown. For each sample, cells in three randomly chosen visual fields (magnification was × 10) were counted and averaged. The statistically significant differences between the groups were calculated, and represented as ^∗^*P* ≤ 0.05 values vs. late EPCs-C and ^∗∗^*P* ≤ 0.05 values vs. late EPCs-HH.

The effect of miR223 and IGF-1R was also assessed on migratory capacity of EPCs. The results indicated the transfection efficiency of miR-223 on the migratory ability of both late-EPCs-C and late-EPCs-HH, since the number of transmigrated cells was enhanced by ∼1.98-fold for C cells, and 2.20-fold for HH cells, compared to late EPCs alone (*P* ≤ 0.01, Figure 9). Similarly, IGF-1R co-incubation induced an augmentation of transmigrated cell number in the Boyden chamber by 1.49-fold for late EPCs-C and 1.77-fold, for late EPCs-HH, respectively (*P* ≤ 0.05, Figure 9).

## Discussion

The present study shows the importance of interplay between EPCs and platelets during atherosclerosis in a *in vitro* model. Specifically, we assessed the impact of platelets on EPC function and we highlighted the role of chemokines, miR-223 and IGF-1R in this process. We have found that interaction with platelets of healthy origins augments the functional properties of late EPCs in experimental induced atherosclerosis. Moreover, we provide evidences that the presence of healthy origin platelets is of utmost importance on functional improvement of EPCs in atherosclerosis by miR-223 transfer and subsequent targeting of the chemokines and IGF-1R.

The studies showing a significant correlation between the EPC levels and functions, and different cardiovascular disease states or risk factors suggest that EPCs represent a group of progenitor cells with a significant impact and clinical relevance (Hill et al., 2003; Dernbach et al., 2008; Fleissner and Thum, 2008; Vaturi et al., 2011; Lee and Poh, 2014; Lev et al., 2014; Alexandru et al., 2017a). Moreover, several studies indicated the essential EPC role in the vascular injury repair, and also the involvement of platelets in EPC recruitment to injury site, in their maturation and differentiation to endothelial cells. In addition, it has been demonstrated that, *in vitro*, platelets stimulate early EPC ability to proliferate, migrate, to form colonies and express endothelial markers (Daub et al., 2006; de Boer et al., 2006; Lev et al., 2006; Massberg et al., 2006; Dernbach et al., 2008; Leshem-Lev et al., 2010). In our previous article, we showed that intravenous administration of circulating healthy EPCs has a helpful effect on EPC-platelet relationship in experimental atherosclerotic model, improving both EPC and platelet functions and reducing inflammatory markers (Alexandru et al., 2015).

These prior studies were focused on circulating EPCs and *in vitro* early EPCs obtained from human subjects. In the current study, we followed the effects exerted by platelets isolated from control healthy hamsters (C) and experimental animal model of atherosclerosis, hypertensive-hyperlipidemic hamsters (HH), on late EPCs obtained from C and HH groups, in the direct co-culture conditions. In our previous study, late EPCs isolated and expanded from peripheral blood drawn from C and HH groups were characterized *in vitro* for their ability to uptake acetylated low-density lipoprotein (acLDL) and ulex europaeus agglutinin 1 (UEA-1) lectin and express endothelial cell markers (Alexandru et al., 2017a). Here in, we demonstrated that *in vitro* exposure to platelets from control hamsters improved the late EPCs-C ability to form colonies and tube-like structures, to proliferate and migrate and improved their functions in atherosclerosis as well. In addition, platelets from HH hamsters reduced the functional capabilities for both late EPCs-C and late EPC-HH. Thus, we can suppose that platelets of healthy origins could increase EPC ability to accelerate re-endothelisation at the injury site, and they also contribute to EPC function improvement in atherosclerosis. By fluorescence microscopy, we showed that platelets can attach to late EPCs in culture condition, the interaction being reduced in atherosclerotic conditions and reinforced when the late EPCs-C or late EPCs-HH were exposed to healthy platelets from C group. These data correlate with the results from our previous study in a dynamic flow system, where we found that circulating EPC binding to platelets was decreased for freshly platelets obtained from HH hamsters compared to those obtained from C group (Alexandru et al., 2015). The activated status of platelets found at our animal model of atherosclerosis (HH hamster) (Alexandru et al., 2013) could explain these results. In addition, the platelet activation in the HH group was demonstrated by the increase of proteic expression of integrin β3 and molecules involved in αIIbβ3 integrin signaling, such as focal adhesion kinase, p85 subunit of PI3-Kinase and Src (Alexandru et al., 2013).

Furthermore, in attempt to explore the potential mechanisms underlying the improvement of late EPC functionality in the presence of platelets, we investigated the levels of several chemokines and growth factors secreted into the culture medium during the 7 days of incubation of platelets with late EPCs.

First of all, we measured the concentration SDF-1α, a chemokine with a major role in the bone marrow derived progenitor cell recruitment and homing to the vascular injury sites (Zernecke et al., 2005). Moreover, it has been revealed that SDF-1α can influence the EPC homing to sites distal of pre-existing vasculature and formation of new vessels, or supporting the propagation of new vessels from pre-existing ones (Asahara et al., 1999a,b; Ceradini et al., 2004; Awad et al., 2006). In the current study, co-incubation of late EPCs, obtained both from C and HH group with platelets from C group, significantly increased the levels of this chemokine in the culture growth medium. The platelet-derived SDF-1α possible role in mediation of platelet effects on EPC function is supported by other studies. Massberg et al. (2006) demonstrated that activated platelets secrete SDF-1α immediately after vascular injury (in the first 30 min), sustaining migration and subsequently, adhesion, of progenitor cells at the vascular injury site. Furthermore, Leshem-Lev et al. (2010) showed that the levels of SDF-1α were higher in the cell culture medium obtained after co-incubation of early EPCs from healthy subjects with isolated platelets, vs. EPCs alone.

Secondary, we explored the pro-angiogenic factors, VEGF and PDGF with main role in the angiogenesis process (Cross and Claesson-Welsh, 2001; Dimmeler, 2005). Also, it has been shown that VEGF contributes to the EPC homing to the site of injury and promotes neovascularization (Asahara et al., 1999a,b). We have found in the current study that VEGF and PDGF-AB isoform concentrations were greater in medium from EPCs co-incubated with platelets from C group, in comparation with EPC alone. These results are in concordance with a recent study showing that PDGF has a beneficial effect on EPCs and a central role in the improvement in EPC functional properties in response to platelets (Raz et al., 2014).

Additionally, the levels of pro-inflammatory mediators (CD40L and IL-1β,-6,- 8), with major role in the atherosclerosis development, were measured in culture medium (Lievens and von Hundelshausen, 2011).

We found that the co-incubation with platelets of healthy origins reduced the levels of these factors, indicating that EPC exposure to platelets in culture contributes to reducing the inflammatory microenvironment generated in medium from late EPCs isolated from atherosclerotic hamsters. The incubation with the platelets from HH group had a contrary effect on these pro-angiogenic and pro-inflammatory molecules for both cultures, late EPCs-C and late EPCs-HH.

As it has been shown that miRNAs have a significant contribution to cardiovascular physiology and pathology, and their abnormal expression has been correlated with atherosclerosis, in this study we have decided to analyze the miR-223, a miRNA with an essential role in platelet function (Fuentes et al., 2015). Besides, profiling studies revealed that miR-223 has an increased expression in normal endothelial cells and an important contribution in vasculogenesis (Shi et al., 2013). Our data demonstrated that dysfunctional late EPCs from HH group have lower miR-223 levels then late EPCs-C, and their incubation with platelets of healthy origins significantly increased miR233 levels. The co-incubation with platelets from HH group does not have the same effects. In addition, we have demonstrated that platelets from C group increased the expression of IGF-1R, one of the miR-223 target, in late EPCs-HH. The IGF system, including also IGF-1R, plays the pivotal role in normal growth and development of the endothelial cells, by promoting migration, tube formation and NO production (Bayes-Genis et al., 2000; Bach, 2015). Moreover, it has been showed that IGFs increase the EPC number and function, and protect against atherosclerosis (Bach, 2015). In accordance with this study our results indicated that miR-223 and IGF-1R directly improve late EPC functional properties in atherosclerosis by stimulating the proliferation and migration abilities.

Therefore, our data suggest that the positive effects of healthy platelets on late EPC function in atherosclerosis can be explained by increasing miR-223 levels that target IGF-1R as results of their transfer from platelets. This transfer may be also mediated by MPs released by platelets, since it is known that MPs are carriers for various miRNAs, related with cardiovascular diseases (Diehl et al., 2012; Alexandru et al., 2017b). Moreover, MPs are involved in intercellular communication, and several data indicated that by delivering RNAs, they can affect protein expression of their target cells (Diehl et al., 2012; Gidlof et al., 2013). Thus, it has been shown that miR-320b secreted by platelets can be taken up by endothelial cells and control intercellular adhesion molecule (ICAM-1) gene expression (Gidlof et al., 2013).

Our results bring new data regarding positive effects of healthy origin platelets on late EPC dysfunctions associated with atherosclerosis. Moreover, we have found that this improvement can be mediated by secreted chemokines and factors originating from platelets or EPCs, such as SDF-1α, VEGF, CD40L, PDGF, and IL-1β,-6,-8, and also by miR-223 transfer into the target cells, regulating the IGF-1R expression. Taken together, our findings contribute to understanding the mechanisms involved in the EPC-platelet interaction, with a central role in process of repair following vascular injury. Also, our data may support the progress in the development of new approaches to improve EPC function in patients with cardiovascular diseases. The understanding that EPCs act in concert with other cells will lead to new perspectives of their use, both for repair and maintenance of existing vasculature within the body as targets for endothelialization of implantable devices required for the treatment of cardiovascular diseases as well. However, continued research is required to provide valuable data to guide the efforts toward the rational design for a new therapeutic approach of this cellular target.

In addition to the well-documented roles of interaction of EPCs with platelets at the sites of vascular injury mainly within the thrombi and along the vessel wall, our findings highlight a new biological role for platelets in regulating EPC function via SDF-1α, VEGF, CD40L, PDGF, IL-1β,-6,-8 chemokines, miR-223, and IGF-1R in atherosclerosis. Eventually, this may lead to the development of novel therapies based on targeting interplay between platelets and EPCs in patients with cardiovascular disease.

### Reagents

The antibodies, primers, TaqMan kits, culture medium, reagents, were acquired from Thermo Fisher Scientific (United States), Santa Cruz Biotechnology (United States), Qiagen (Germany), R&D Systems (United States), Merk & Co. (United States), Lonza (Switzerland), and Sigma Chemical Co. (St. Louis, MO, United States).

### Data Analysis

To quantify and compare the data, One-Way ANOVA method and Bartlett’s test in GraphPad Prism 7.02 program were applied.

The statistically significant differences between the groups were calculated, and represented as *P* ≤ 0.05 values.

## Ethics Statement

All the experimental procedures have been approved by the Ethics Committees from Institute of Cellular Biology and Pathology “Nicolae Simionescu,” and they have been achieved in accordance with National, European and International legislation on the use of experimental animals in biomedical research (Alexandru et al., 2017a).

## Author Contributions

AG and NA contributed to the conception and design of the study. NA, FS, AC, MN, GT, and AF performed the experiments, contributed to acquisition, analysis, and interpretation of the data. ED effectively participated at the realization of the experimental models. AG and EB substantially contributed to the analysis and interpretation of the data, and critically revised the manuscript for the important intellectual content. NA, FS, and AC wrote the drafted manuscript.

## Conflict of Interest Statement

The authors declare that the research was conducted in the absence of any commercial or financial relationships that could be construed as a potential conflict of interest.

## Abbreviations

BSA: bovine serum albumin
CD40L: CD40 ligand
DAPI: 4′,6-diamidino-2-phenylindole
ELISA: enzyme-linked immunosorbent assay
EPCs: endothelial progenitor cells
FBS: fetal bovine serum
FITC: fluorescein isothiocyanate
IGF-1R: insulin-like growth factor 1 receptor
IL-1β,-6,-8: interleukins-1β,-6,-8
KDR: kinase insert domain receptor
MNCs: mononuclear cells
MPs: microparticles
MTT: 3-(4,5-dimethylthiazol-2-yl)-2,5-diphenyltetrazolium bromide
PBS: phosphate-buffered saline
PDGF: platelet derived growth factor
PFA: paraformaldehyde
PRP: platelet-rich plasma
SDF-1α: stromal-cell-derived factor-1
VEGF: vascular endothelial growth factor
VEGFR2: vascular endothelial growth factor receptor 2
vWF: von Willebrand factor.
