# Supplementary material for: Platelets of Healthy Origins Promote Functional Improvement of Atherosclerotic Endothelial Progenitor Cells
Source: Front Pharmacol. 2019 Apr 24;10:424. doi: 10.3389/fphar.2019.00424 (PMC6491786; doi:10.3389/fphar.2019.00424)
Supplement: Supplementary file 1 [file Data_Sheet_1.doc]

***Isolation and characterization of late EPCs***

These procedures were performed according to previously described methods by [Alexandru et al., 2017a](#_ENREF_2).

Briefly, total mononuclear cells (MNCs) were isolated from peripheral blood collected in tri-sodium citrate anticoagulated tubes, using Histopaque-1077 density-gradient centrifugation (400xg, 30min, at 24°C). The opaque interface containing MNCs was transferred into clean tube and after washing with phosphate buffered saline solution (PBS) by centrifugation at 250xg for 10min, was treated with ACK lysing buffer, and again washed with PBS by centrifugation at 250xg for 10min. Finally, MNCs were resuspended in endothelial cell basal medium-2 (EBM-2) culture medium enriched with endothelial growth medium (EGM-2 SingleQuots) and growth factors (Lonza) (EBM-2-EGM-2) and cultured at a density of 1×10^6^ cells/500µl on collagen type I-coated 4-well plates in EBM-2-EGM-2 for 4 weeks to generate the late EPC culture. After 3 days of culture, the EBM-2-EGM-2 medium containing the non-adherent cells and debris was discarded, the cells were washed with EBM-2 medium and finally a new medium (EBM-2-EGM-2+10% FBS (fetal bovine serum)) was added. The latter medium was changed to 2 days for 4 weeks when the late EPC were obtained. When late EPCs were obtained from MNCs isolated from peripheral blood of C group they were called **late EPCs-C**, and when late EPCs were obtained from MNCs isolated from peripheral blood of HH group they were called **late EPCs-HH.**

In distinct experiments, the late EPCs were characterized by dual-staining for 1,1-dioctadecyl-3,3,3,3 tetramethylindocarbocyanine-labeled acetylated low-density lipoprotein (Dil-Ac-LDL) and fluorescein-isothiocyanate (FITC)-conjugated Ulex europaeus agglutinin lectin (UEA-1). For this, the 2x10^4^ EPCs were incubated with Dil-Ac-LDL (6µg mL^-1^) at 37°C for 2h, in darkness, washed 2 times with PBS and subsequently fixed with 1% paraformaldehyde (PFA) for 10min. Then the cells were reacted with UEA-1 (10mg L^-1^) at 37°C for 2h. After the staining and mounting with Fluoroshield with DAPI, the samples were viewed under an inverted fluorescent microscope (Axio Vert.A1 Fl, Carl Zeiss, software Axio Vision Rel 483SE64-SP1). The cells double stained for both UEA-1 and Dil-Ac-LDL as late EPCs were taken into account (see [Alexandru et al., 2017a](#_ENREF_2)).

Also, the late EPCs were identified by demonstrating the presence on their surface of CD34, CD133, KDR, CD144, vWF, Tie-2 markers, and the absence of CD14, CD45. Concisely, after tripsinization with 0.25% trypsin for 30min, pelleted EPCs were resuspended in EBM-2-EGM-2 medium and incubated for 40 min at room temperature (RT) in darkness with specific antibodies for the above markers. The percentages of stained EPCs were determined using flow cytometric analysis (see [Alexandru et al., 2017a](#_ENREF_2)).
